# Editorial: Reviews in educational psychology

**DOI:** 10.3389/fpsyg.2025.1647910

**Published:** 2025-08-04

**Authors:** Alberto Díaz-Burgos, Jesús N. García-Sánchez, María-Lourdes Álvarez-Fernández, Jesús de la Fuente, Douglas F. Kauffman, Ting-Chia Hsu

**Affiliations:** ^1^Department of Psychology, Sociology and Philosophy, Universidad de León, León, Spain; ^2^Department of Psychology, School Education and Psychology, University of Navarra, Pamplona, Spain; ^3^School of Clinical Medicine, Medical University of America-Nevis, Devens, MA, United States; ^4^Department of Technology Application and Human Resource Development, National Taiwan Normal University, Taipei City, Taiwan

**Keywords:** reviews in educational psychology, systematic review of reviews, systematic reviews, scoping review, meta-analysis

## 1 Introduction

The present article introduces the Research Topic “*Reviews in educational psychology*,” part of the “Reviews in” series launched by Frontiers in Psychology. This initiative highlights the growing importance of synthesis studies in consolidating knowledge and advancing the maturity of the discipline (Campos et al., 2024; Jaramillo-Mediavilla et al., 2024).

Educational Psychology, as an applied field, has reached a stage where synthesizing accumulated evidence is not only valuable but necessary. This collection showcases the diversity of review formats—systematic, theoretical, scoping, meta-analytical—and includes a comprehensive systematic review of reviews, reflecting a shift toward second-order syntheses that integrate large bodies of prior research.

Although the focus is on reviews, a small number of original studies are also featured. These contributions, some based on expert consensus or large-scale data analysis, complement the review findings by offering empirical insights into key constructs and emerging research trends.

**Figure 1:**
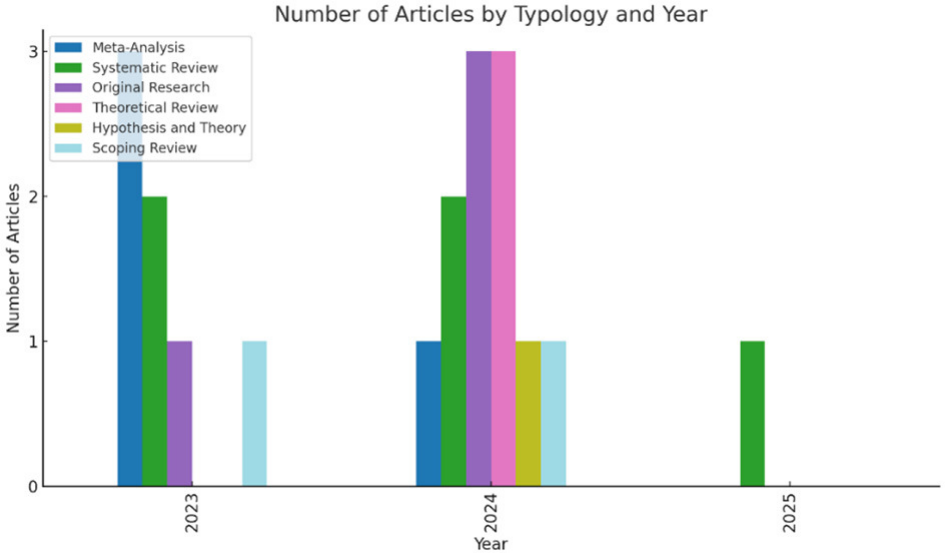
Articles published in Special Research Topic by Typology and Year.

The distribution of articles by typology and year illustrates both the scope of contributions and the growing methodological variety in the field. Together, these works demonstrate how diverse approaches—qualitative, quantitative, and mixed—contribute to a cumulative, theory-informed understanding of psychological processes in education (DeCuir-Gunby and Schutz, 2024; Kumar and DeCuir-Gunby, 2023), as summarized in Figure 1.

## 2 Scientific contributions

### 2.1 Reviews

In a context where the number of systematic reviews and meta-analyses has grown exponentially, a new layer of synthesis is necessary to organize the accumulated knowledge. This is precisely the aim of Díaz-Burgos et al., who conducted a systematic review of reviews encompassing 392 review studies focused on digital education and the Sustainable Development Goals (SDGs). Their work not only identifies common review typologies, regional gaps, and emerging digital tools, but also highlights the relevance of structured strategies such as PICOC or SALSA in guiding transparent and rigorous synthesis processes. By mapping current trends and research voids, this study reinforces the value of second-order syntheses as essential tools to support inclusive policies and sustainable educational practices.

Following this, several systematic reviews address key psychological constructs from diverse perspectives. Kuznetsova et al. synthesized 104 studies on giftedness, identifying cognitive, physiological, and psychological traits—especially strong performance in motivation and executive functioning—that distinguish gifted students. Taking a more experimental focus, Wang S et al. reviewed 53 studies on fixation in problem-solving and creativity, concluding that overcoming fixation substantially enhances performance in closed-ended tasks. In another vein, Wu et al. examined 18 studies on student anxiety during the COVID-19 pandemic, showing that physical activity and mindfulness-based strategies are highly effective in reducing stress. Additionally, Yu synthesized 49 studies on foreign language anxiety, primarily in English as a Foreign Language (EFL) contexts, and emphasized the need for broader methodological and geographical diversity in future research.

Theoretical reviews in this issue contribute valuable conceptual advances. Bonilla-Sánchez explored neurodevelopmental disorders through qualitative neuropsychological assessment, offering a culturally sensitive alternative to traditional diagnostic models. Similarly, Stoltz et al. compared theories of consciousness development by Piaget, Vygotsky, and Steiner, integrating developmental, cultural, and holistic perspectives to enrich contemporary educational theory. In a more applied domain, Wang D and Li conducted a mini-review on career construction theory, highlighting assessment tools such as narrative methods, interviews, and digital interventions, with implications for increasingly personalized professional guidance.

Scoping reviews in this monograph examine structural and psychological aspects in doctoral experience. Hurtado et al. identified, across 32 studies, multiple factors influencing doctoral student retention—including individual, academic, socioeconomic, and institutional dimensions—proposing a multifactorial approach. In a related line, Wang Y and Li reviewed 30 studies on the impostor phenomenon among doctoral students, revealing its widespread prevalence and its impact on academic performance and psychological wellbeing. Both studies underscore the urgent need for tailored institutional support to ensure success in advanced academic stages.

Finally, the collection includes several meta-analyses that provide robust evidence on impactful educational interventions. Cochon Drouet et al. analyzed 43 studies on the Jigsaw method, identifying heterogeneous effects on achievement, motivation, and social relations, and offering context-sensitive implementation guidelines. Fan et al. conducted a meta-analysis of 30 studies examining parental involvement in student creativity, reporting a modest yet significant positive effect moderated by factors such as age and cultural background. Also focusing on learning processes, Shao et al. synthesized 46 studies on scaffolding in self-regulated learning (SRL), highlighting the effectiveness of composite tools and intelligent pedagogical agents. Zheng et al. explored the role of emotions in SRL through a meta-analysis of 23 studies, proposing a multimodal framework where positive emotions act as facilitators and negative emotions as barriers to effective self-regulation strategies. Finally, de la Fuente and Martínez-Vicente proposed a theoretical model linking stress and psychological wellbeing, outlining predictive, mediating, and functional components that may inform future intervention studies or theoretical syntheses (Table 1).

**Table 1:** Overview of *Reviews in educational psychology*.

**Paper**	**Year**	**Country**	**N**	**Construct**	**Objectives**	**Results**	**Add value**
**Systematic reviews of reviews**							
Díaz-Burgos et al.	2025	Spain, Portugal, USA, Taiwan	392 reviews	Review methodology, digital education, SDGs	Synthesize review trends and gaps in educational psychology	Identifies typologies, regions, and digital evolution patterns	Supports inclusive and sustainable review-based research practices
**Systematic reviews**							
Kuznetsova et al.	2024	Finland, Russia, Canada	104 studies	Giftedness, intelligence, cognitive traits	Compare gifted vs. non-gifted children's characteristics	Gifted outperform peers in cognition, motivation, brain activity	Useful for developing assessments and gifted education programs
Wang S et al.	2023	Japan	53 studies	Fixation in creativity/problem solving	Analyze fixation types, sources, and effective defixation methods	Fixation impedes closed problems; within-frame helps open problems	Encourages tailored strategies to overcome creativity blocks effectively
Wu et al.	2023	Malaysia, China	18 studies	COVID-19 impact on student anxiety	Assess pandemic-induced anxiety and effectiveness of interventions	Physical activity and mindfulness effective; information reduces anxiety broadly	Encourages targeted coping strategies for sustained mental health interventions
Yu	2024	China	49 studies	Foreign language anxiety (FLA)	Review trends, methodologies, and findings in FLA research	EFL context dominant; mainly quantitative and mixed methods; high Chinese representation	Suggests future focus on diverse contexts and qualitative explorations
**Theoretical reviews**							
Bonilla-Sánchez	2024	Mexico	Not specific	Neurodevelopmental disorders and school learning	Present clinical cases of qualitative neuropsychological intervention	Qualitative assessment identifies strengths, weaknesses, and dynamic brain functions	Alternative to classical models using cultural–historical neuropsychology
Stoltz et al.	2024	Brazil, Germany	Not specific	Consciousness development	Compare educational models of consciousness development	Integrates developmental, cultural, holistic perspectives	Enriches educational theory with integrative theoretical models
Wang D and Li	2024	China	22 studies	Career construction theory tools and interventions	Present assessment tools, interventions, and future trends for career counseling	Effective tools include interviews, narrative methods, digital counseling strategies	Proposes further research on digital interventions and standardization
**Scoping reviews**							
Hurtado et al.	2024	Spain, Colombia	32 studies	Doctoral student retention factors	Identify factors affecting doctoral student retention and dropout	Individual, academic, socioeconomic, institutional factors interact	Proposes multifactor approach for doctoral student permanence
Wang Y and Li	2023	China	30 studies	Impostor Phenomenon among doctoral students	Review research on characteristics, impacts, measurement of impostor phenomenon	Impostor feelings prevalent, impact academic and psychological outcomes	Emphasizes need for targeted support and measurement refinement
**Meta-analysis**							
Cochon Drouet et al.	2023	Switzerland	43 studies	Achievement, motivation, social relations, self-esteem	Analyze effects of Jigsaw method on educational outcomes	Heterogeneous effects found across learning and social indicators	Provides implementation guidelines and identifies moderating variables
Fan et al.	2024	China	30 studies	Parental involvement and student creativity	Analyze parental involvement's effect on creativity in students	Small but significant positive effect on creativity development	Highlights moderators like age, cultural background, and involvement type
Shao et al.	2023	China	46 studies	Self-regulated learning, SSRL, scaffolding	Analyze effects of learning scaffolding on regulation and performance	Scaffolding improves strategies, composite tools most effective	Advises diverse scaffolding for SRL and collaborative learning
Zheng et al.	2023	USA, Canada	23 studies	Emotions in self-regulated learning (SRL)	Investigate role of trait and state emotions in SRL framework	Proposes integrated framework; positive emotions facilitate effective SRL strategies	Recommends multimodal methods for deeper emotion–SRL relationship understanding
**Hypothesis and theory**							
de La Fuente and Martínez-Vicente	2024	Spain	Not specific	Stress and psychological wellbeing	Propose conceptual model for stress and wellbeing management	Defines predictive, mediating and final functional model factors	Applicable across education, health, organizational and digital contexts
**Original researches**							
López Martínez et al.	2024	Spain	16 experts	Verbal creativity indicators in writing tasks	Identify valid indicators for assessing verbal creative thinking	Indicators include fluency, originality, elaboration, flexibility, refinement	Provides assessment criteria for verbal creativity in education
Romero-González et al.	2023	Spain	54 participants	Home Literacy Environment, motivation, affective bonds	Assess effects of active Home Literacy Environment program	Program enhances reading, motivation, family relationships	Highlights importance of active family-school collaboration
Ünal et al.	2024	Finland	Not specific	Teacher values and emotions (rational/non-rational truth)	Examine relationships between values, truth types, and teacher emotions	Non-rational truth linked to anxiety; rational truth to self-direction/enjoyment	Highlights value-emotion link implications for teacher wellbeing
Wang et al.	2024	China	1,638 articles	Early reading trends via dynamic topic modeling	Analyze early reading research trends and topic evolution over time	Identified 11 topics; foundational skills and autism notably increased	Highlights emerging research directions, aiding future academic inquiries

Analyses were primarily conducted using the PRISMA methodology, with the exception of Theoretical Reviews, Hypothesis and Theory and Original Researches.

Although the main focus of this Research Topic lies in review studies, several original research articles have been included due to their strong thematic alignment with the constructs and challenges identified through synthesis work. López Martínez et al. employed a Delphi method with 16 international experts to identify key indicators of verbal creativity—such as fluency, originality, and elaboration—thus offering assessment criteria that can guide future empirical research and systematic inquiry. Similarly, Romero-González et al. evaluated an active Home Literacy Environment program for children aged 6 to 8, demonstrating significant improvements in reading motivation, family relationships, and academic outcomes. While not reviews, these studies contribute grounded, context-rich evidence to key educational psychology domains addressed in the broader collection.

Other original studies in the monograph provide conceptual or methodological contributions that complement the review-based focus. Ünal et al. examined Turkish teachers' personal values and emotional responses to rational and non-rational truths, offering novel insights with implications for teacher training and professional wellbeing. Wang T et al. conducted a large-scale bibliometric analysis of 1,638 articles using dynamic topic modeling to identify evolving themes in early reading research—an approach that, while empirical, serves to map trends typically targeted by scoping reviews.

## 3 Conclusions

Collectively, these contributions reflect the evolving and multifaceted landscape of educational psychology research, highlighting critical issues such as creativity, emotional regulation, literacy, teacher values, and mental health in education. Each study enriches our understanding and provides practical implications, ultimately guiding educators, researchers, and policymakers toward more informed and effective practices. This compilation underscores the necessity for ongoing interdisciplinary collaboration, robust research methodologies, and continuous innovation to address educational challenges in an ever-changing global context.
